# *N*‐butylphthalide (NBP) ameliorated ischemia/reperfusion‐induced skeletal muscle injury in male mice via activating Sirt1/Nrf2 signaling pathway

**DOI:** 10.14814/phy2.70149

**Published:** 2024-11-30

**Authors:** Peng Lu, Wei‐Peng Li, Ben‐Jun Zhou, Wen‐Ze Tian, Xiang Lu, Wei Gao

**Affiliations:** ^1^ Department of Geriatrics Sir Run Run Hospital, Nanjing Medical University Nanjing China; ^2^ Department of Geriatrics The Affiliated Huai'an No.1 People's Hospital of Nanjing Medical University Huai'an China; ^3^ Department of Cardiology The Second Affiliated Hospital of Nanjing Medical University Nanjing China; ^4^ Department of Thoracic Surgery The Affiliated Huai'an No.1 People's Hospital of Nanjing Medical University Huai'an China; ^5^ Department of Geriatrics Zhongda Hospital, School of Medicine, Southeast University Nanjing China

**Keywords:** ischemia/reperfusion, *N*‐butylphthalide, Nrf2, Sirt1, skeletal muscle

## Abstract

*N*‐butylphthalide (NBP) has been reported to have potential protective effects in ischemic stroke via its antioxidative properties. The present study was aimed to investigate the protective effects of NBP on ischemia/reperfusion (I/R)‐induced skeletal muscle injury. Mouse model of I/R‐induced skeletal muscle injury and hypoxia/reoxygenation (H/R)‐induced C2C12 myotube injury model were constructed to test the protective effects of NBP both in vivo and in vitro. Our results showed that I/R resulted in skeletal muscle injury, as evidenced by elevated levels of LDH, CK, ROS, 3‐NT, MDA, and 4‐HNE as well as decreased activities of SOD, GSH‐Px, and decreased expression of Myog and MyoD in gastrocnemius muscle, which was ameliorated by NBP treatment. Mechanistically, NBP treatment increased the expression of Sirt1 and Nrf2 in the injured skeletal muscle. Notably, the protective effects of NBP on I/R‐induced skeletal muscle injury was diminished by the treatment of Sirt1 inhibitor. Further studies in H/R‐induced C2C12 myotubes injury model also showed that NBP activated the Sirt1/Nrf2 pathway. NBP treatment upregulated the expression of myog and MyoD in H/R‐stimulated C2C12 myotubes, which was eliminated by silencing of Sirt1. Taken together, our results suggest that NBP may alleviated I/R‐induced skeletal muscle injury by activating Sirt1/Nrf2 signaling pathway.

## 1 INTRODUCTION

Acute limb ischemia is one of the severe vascular diseases that causes arterial embolism, artery transplantation, trauma, abdominal compartment syndrome, etc. (Gillani et al., 2012). Although interventional approaches for vascular reperfusion can rescue ischemic muscle, reperfusion often induces a burst of reactive oxygen species (ROS) which in turn causes oxidative stress, resulting in additional skeletal muscle injury named ischemia/reperfusion (I/R) injury (Barnig et al., 2022). However, to date, the treatment for skeletal muscle I/R injury is mainly relied on symptomatic supportive alleviation, it is therefore urgent to explore effective therapeutic medications for this common injury.

The exact molecular mechanisms underlying the trigger of oxidative stress during the development of I/R injury in skeletal muscle remain unclear. Silent information regulator of transcription 1 (SIRT1) is a nicotinamide adenosine dinucleotide (NAD) ‐dependent deacetylase, which is involved in maintaining the balance of cell redox (Meng et al., 2017). Sirt1 is located in the cytoplasm and nucleus and has various of interacting proteins, including nuclear factor erythroid‐2 related factor 2 (Nrf2) (Singh & Ubaid, 2020). Nrf2 has been considered as a key factor for the maintenance of skeletal muscle function and plays a crucial role in preventing oxidative stress‐induced injury in skeletal muscle (Wafi et al., 2019). Previous studies have demonstrated that activation of Sirt1/Nrf2 pathway can attenuate skeletal muscle I/R injury (Cheng et al., 2016; Zhang et al., 2019).

DL‐3‐*N*‐butylphthalide (NBP) is a natural compound isolated from *Apium graveolens* seeds (Chinese celery) (Hu et al., 2023). NBP has been approved for the treatment of ischemic stroke by the State Food and Drug Administration of China in 2002. Numerous studies have shown that the neuroprotective effects of NBP may be related to multiple mechanisms including increased regional blood flow, reduced brain edema and neuronal apoptosis, attenuated blood–brain barrier damage, improved mitochondrial function, and decreased oxidative damage and inflammatory response (Wang et al., 2018). Emerging evidence suggests that NBP also has potential beneficial effects on I/R injury of brain (Huang et al., 2021; Qin et al., 2019), heart (Zhang, Zheng, et al., 2022), and renal (Dong et al., 2021). Interestingly, a recent study showed that NBP alleviated limb I/R‐driven muscle tissue damages by suppressing inflammation and oxidative stress via inhibition of HMGB1/TLR4/NF‐κB pathway (Sun et al., 2022). In the present study, we sought to investigate whether NBP treatment could ameliorate I/R‐induced skeletal muscle injury through activating Sirt1/Nrf2 signaling pathway.

## 2 MATERIALS AND METHODS

### 2.1 Animals and treatment

Male 8‐week‐old C57BL/6 mice were obtained from Weitonglihua Experimental Animal Tech Co. (Beijing, China). All mice were housed at a controlled temperature of 24° ± 2°C and relative humidity of 45% ± 15% with a 12‐h light/dark cycle. Mice were unrestricted access to water and a standardized diet (1010001, Xietong Shengwu, China). All animal protocols were in accordance with the ethical standards laid down in the 1964 Declaration of Helsinki and its later amendments and approved by the Institutional Animal Care and Use Committee of Nanjing Medical University (IACUC‐1905027). Firstly, mice were randomized into six groups (*n* = 8) as control, 10 mg/kg NBP, 20 mg/kg NBP, I/R, 10 mg/kg NBP + I/R and 20 mg/kg NBP + I/R. For the treatment of NBP, mice were received intraperitoneal injection of NBP (BD88426, Bide Pharmatech Ltd., China) one day before the surgery. An equivalent volume of 0.9% sodium chloride was injected in the control and I/R model groups. Unilateral left hind limb ischemia was achieved by positioning a tourniquet consisting of an orthodontic rubber band at the hip joint for 3 h, followed by removing the rubber bands for 24 h (Guo et al., 2021). The mice were euthanized using sevoflurane anesthesia followed by cervical dislocation. Gastrocnemius muscles of the left limbs were harvested. For the treatment of EX527, mice were randomized into three groups (*n* = 8) as I/R, NBP + I/R, NBP + I/R + EX527. Mice were received intraperitoneal injection of 10 mg/kg EX527 (HY‐15452, MedChemExpress, USA) in NBP + I/R mice 30 min before the administration of NBP. An equivalent volume of 0.9% sodium chloride was injected in the I/R and NBP + I/R groups.

### 2.2 C2C12 culture and treatment

Murine C2C12 myoblasts were obtained from ATCC and incubated at 37°C, 5% CO_2_ in DMEM (KGL1211‐500, KeyGen BioTECH, China) with 80 U/mL penicillin and 0.08 mg/mL streptomycin (15140122, Thermo Fisher Scientific, USA) and 10% fetal bovine serum (A5670701, Gibco, Thermo Fisher Scientific, USA). When the C2C12 myoblasts reached 80% confluence, cells were moved to differentiation medium containing 2% horse serum (16050122, Gibco, Thermo Fisher Scientific, USA), and then cultured for 4–6 days. For the construction of hypoxia/reoxygenation (H/R) model, myotubes were cultured in hypoxic condition (1% O_2_) for 3 h, and then placed in an incubator with 5% CO_2_ balanced with air at 37°C for 24 h. For the treatment of NBP, myotubes were treated with 10 μM NBP, 24 h in advance. To inhibit Sirt1, myotubes were transfected with 50 nM specific siRNAs (siG09416111955‐1‐5, RiboBio, China) using Lipofectamine™ RNAiMAX (13778030, Thermo Fisher Scientific, USA) for 24 h.

### 2.3 Cell viability

C2C12 cells were seeded in a 96‐well plate at a density of 1 × 10^4^ cells per well and induced to differentiate into mature myotubes. Mature myotubes were treated with increasing doses of NBP (1, 10, 100 μM) for 24 h. Similarly, mature myotubes were treated in advance for 24 h using the above doses of NBP, and then the myotubes were incubated in H/R conditions. CCK‐8 reagent (C0037, Beyotime, China) was added to each well, and after 2 h of incubation, absorbance was measured at 450 nm using a microplate reader (Synergy H1, BioTek, USA). Relative cell viability was calculated according to the instructions.

### 2.4 Assessment of skeletal muscle damage

The serum concentrations of lactate dehydrogenase (LDH) and creatine kinase (CK) were measured spectrophotometrically with commercial assay kits (A020‐1‐2, A032‐1‐1, Nanjing Jiancheng Bioengineering Institute, China) according to manufacturer's instructions.

### 2.5 Assessment of oxidative stress

Glutathione peroxidase (GSH‐Px) and superoxide dismutase (SOD) activities, along with malondialdehyde (MDA) content and ROS levels, were measured spectrophotometrically in gastrocnemius muscle, serum, using commercial kits (A005‐1‐2, A001‐1‐2, A003‐1‐2, E004‐1‐1; Nanjing Jiancheng Bioengineering Institute, China). The levels of 3‐nitrotyrosine (3‐NT) in gastrocnemius muscle as well as serum were measured using commercial ELISA kits (E‐EL‐0040, Wuhan Elabscience Biotechnology Co., Ltd). All assays were conducted strictly following the protocols recommended by the manufacturers.

### 2.6 Hematoxylin–eosin (H&E) staining

Fresh gastrocnemius samples were fixed with 4% paraformaldehyde overnight and embedded with paraffin, serially sliced to 4 μm for H&E staining.

### 2.7 Protein extraction and western blot analysis

Myotubes or gastrocnemius samples were prepared by washing cells with PBS equilibrated to 4°C followed by lysis buffer. An equal amount of protein was separated by 4%–20% SDS‐PAGE (GenScript Biotechnology, Nanjing, China), transferred to PVDF membranes (ISEQ00010, Millipore, Billerica, MA, USA), and then blocked with 5% nonfat milk. Membranes were incubated with specific primary antibodies and the horseradish peroxidase‐conjugated secondary antibody. β‐actin was used as internal control. Enhanced chemiluminescence reagents (34580, Thermo Fisher Scientific, MA, USA) were used to visualize images of the blots. Images were acquired using ChemiDocTM XRS + Imaging System (Bio‐Rad, USA). Band densitometry measurements were assessed using Image Lab 6.0 software. The following primary antibodies were used: Myog (F5D, Developmental Studies Hybridoma Bank, USA), MyoD (sc‐32,758, Santa Cruz Biotechnology, USA), 4‐hydroxynonenal (4‐HNE, ab46545, abcam, USA), Sirt1 (9475S, Cell Signaling Technology, USA), Nrf2 (12721, Cell Signaling Technology, USA) and β‐actin (66009‐1‐Ig, Proteintech, China). The following secondary antibodies were used: Anti‐mouse (SA00001‐1, Proteintech, China), Anti‐rabbit (SA00001‐2, Proteintech, China).

### 2.8 Immunofluorescence and fiber cross‐sectional area measurement

Cryosections of skeletal muscle (6 μm) were fixed with 4% paraformaldehyde for 20 min, followed by permeabilization using 0.5% Triton X‐100 for 15 min, and blocking with 5% bovine serum albumin (BSA) for 1 h. The sections were then incubated overnight at 4°C with an anti‐laminin primary antibody (1:300, L9393, Sigma–Aldrich, USA). To visualize the staining, a Cy™3 AffiniPure Donkey Anti‐Mouse IgG (H + L) secondary antibody (1:500, 715‐165‐150, Jackson ImmunoResearch, USA) was applied. Nuclei were counterstained with 4',6‐diamidino‐2‐phenylindole (DAPI, 1 μg/mL, Sigma–Aldrich, USA). Images were captured using a Zeiss Axio Scope fluorescence microscope (Germany). Fiber cross‐sectional area were quantified using the ROI manager plugin of ImageJ software (National Institutes of Health, USA), based on 4–8 images per muscle and 2–3 muscles per condition.

### 2.9 Immunofluorescence staining

C2C12 myotubes were washed with PBS, fixed with 4% paraformaldehyde for 20 min, permeabilized with 0.5% Triton X‐100 for 15 min, and then blocked with 5% BSA for 1 h. Cells were incubated with anti‐MHC (1:200, MF20, Developmental Studies Hybridoma Bank, USA) overnight at 4°C, then incubated with secondary antibody Cy3‐AffiniPure Rabbit Anti‐Mouse IgG (H + L) (1:500, Jackson ImmunoResearch, USA). Nuclear counterstaining was performed with DAPI (1 μg/mL, Sigma–Aldrich, USA). Images were captured using a fluorescence microscope (Zeiss Axio Scope, Germany). Myotube diameters were quantified by randomly selecting 8–10 fields, with 8–10 myotubes measured per field. The myotube diameters were measured using ImageJ software. For each myotube, an average diameter was calculated based on 8–10 measurements taken along its length.

### 2.10 Statistical analysis

Data are presented as mean ± standard deviation (SD) unless otherwise specified. Statistical differences were assessed using unpaired Student's two‐tailed *t*‐tests for two groups and a one‐way ANOVA for three groups or more. Post‐hoc analysis was performed using Tukey's HSD to identify significant differences between specific groups following one‐way ANOVA. A *p* value of <0.05 was considered significant.

## 3 RESULTS

### 3.1 NBP alleviates I/R‐induced skeletal muscle injury in mice

**FIGURE 1:**
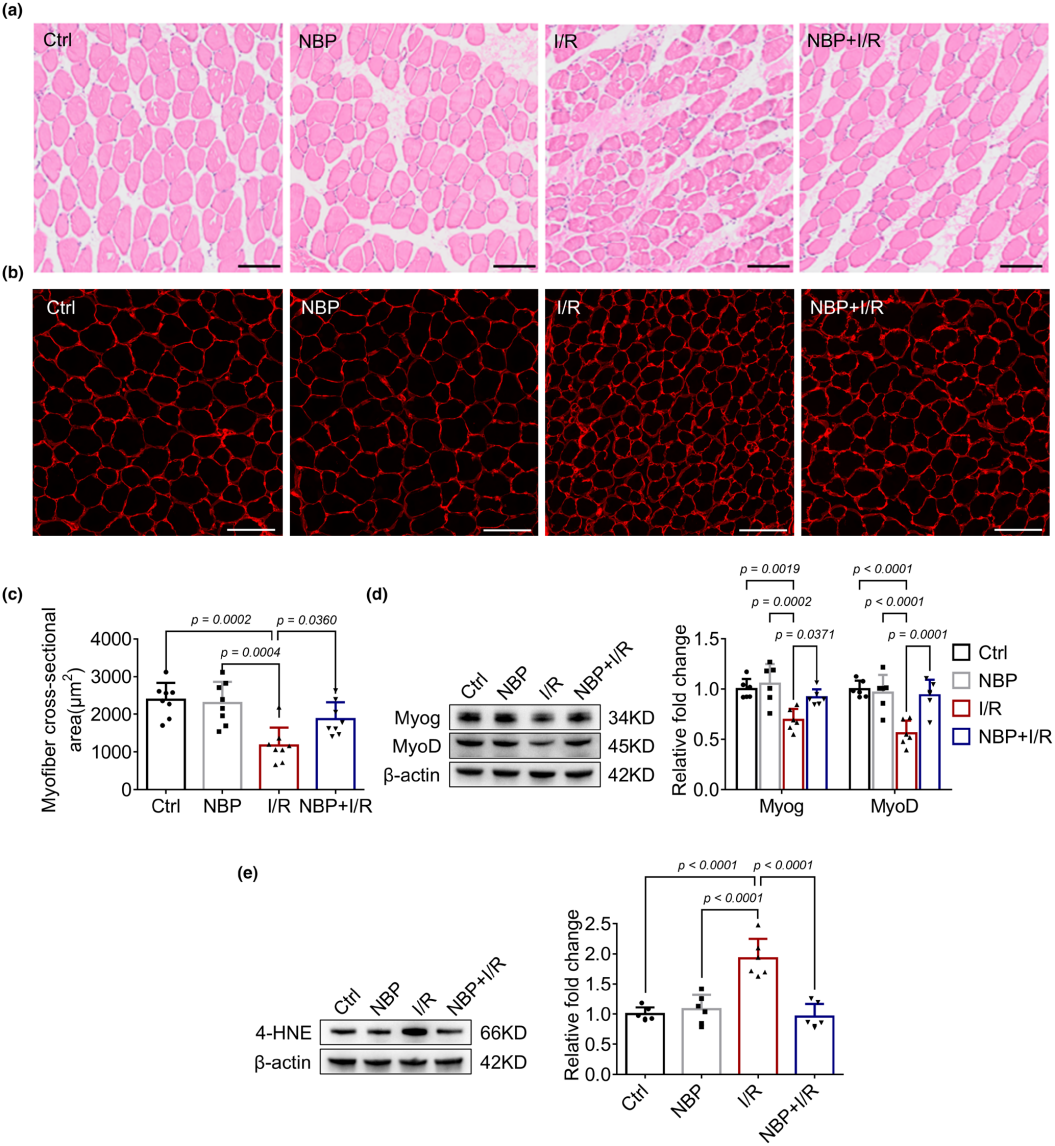
NBP alleviates I/R‐induced skeletal muscle injury. (a–c) H&E staining and immunofluorescence staining of the gastrocnemius muscle indicate the extent of muscle fiber injury and changes in muscle fiber cross‐sectional area across different groups. *n* = 8 per group. Scale bar = 100 μm. A microscope with a 40× objective was used to capture the images. (d) Western blot analysis in gastrocnemius muscle indicates the effects of NBP on Myog, MyoD after I/R. Data are presented as relative fold change to the control group. *n* = 6 per group. (e) Western blot analysis in gastrocnemius muscle indicates the effects of NBP on 4‐HNE expression. Data are presented as relative fold change to the control group (set to 1). *n* = 6 per group.

To determine the protective effect of NBP against skeletal muscle injury, we first constructed a mouse model of I/R‐induced skeletal muscle injury. As shown in Figure 1a, I/R procedure resulted in obvious structural disorder of skeletal muscle fibers, as well as decreased cross‐sectional area of the muscle fibers (Figure 1b,c) and expression of myogenic factors Myog and MyoD (Figure 1d), indicating significant skeletal muscle injury. In contrast, treatment with NBP mitigated the I/R‐induced irregularities in muscle fibers, resulting in an upregulation of the cross‐sectional area of the muscle fibers and increased expression of Myog and MyoD in the gastrocnemius muscle. These results suggested that NBP could ameliorate I/R‐induced skeletal muscle injury.

### 3.2 NBP alleviates oxidative stress induced by skeletal muscle I/R injury in mice

Oxidative stress plays a crucial role in the pathogenesis of I/R‐induced injury. Therefore, we investigated the effects of NBP on the I/R‐induced oxidative stress in the skeletal muscle. As shown in Table 1, serum levels of LDH and CK were significantly elevated compared to the control group, indicating muscle breakdown and injury. Furthermore, increased levels of ROS, 3‐NT, and MDA, along with decreased activities of GSH‐Px and SOD, suggesting a disruption of redox homeostasis. NBP treatment attenuated I/R‐induced oxidative stress, as evidenced by decreased levels of ROS, 3‐NT and MDA as well as increased activities of SOD and GSH‐Px in both NBP + I/R group when compared to the I/R group. Similar results were observed in the gastrocnemius muscle of mice (Table 1). Since no superior therapeutic effect was observed with 20 mg/kg NBP compared to 10 mg/kg, the 10 mg/kg dose was selected as the intervention dose based on these results. Moreover, we observed a significant increase in the expression of the oxidative stress biomarker 4‐HNE (a lipid peroxidation product) within the gastrocnemius muscles of mice from the I/R group, which was attenuated following NBP treatment (Figure 1e). These findings suggested that NBP could ameliorate I/R‐induced oxidative stress in the skeletal muscle.

**TABLE 1:** NBP attenuates oxidative stress in mice model of I/R‐induced skeletal muscle injury.

Parameters	Ctrl	10 mg/kg NBP	20 mg/kg NBP	I/R	10 mg/kg NBP + I/R	20 mg/kg NBP + I/R
Serum						
LDH (U/mL)	0.55 ± 0.19	0.58 ± 0.13	0.59 ± 0.13	1.38 ± 0.38**	0.84 ± 0.28^****^	0.81 ± 0.32^****^
CK (U/mL)	0.67 ± 0.15	0.64 ± 0.13	0.59 ± 0.10	1.54 ± 0.41**	1.14 ± 0.33^****^	0.98 ± 0.53***
ROS (U/mL)	456.12 ± 54.28	425.36 ± 69.74	398.76 ± 60.23	765.43 ± 68.98**	478.42 ± 92.74***	488.83 ± 105.52***
3‐NT (ng/mL)	35.84 ± 7.89	38.77 ± 6.83	36.74 ± 7.21	58.53 ± 10.24*	38.77 ± 9.35***	48.72 ± 14.32
MDA (nmol/mg)	4.74 ± 2.51	4.59 ± 1.30	4.48 ± 1.56	8.59 ± 3.13**	6.31 ± 1.05***	5.50 ± 1.29***
SOD (U/mg)	25.43 ± 5.76	26.65 ± 4.78	30.44 ± 6.87	16.53 ± 8.12*	26.88 ± 6.23***	22.11 ± 8.55***
GSH‐Px (U/mg)	214.34 ± 37.25	243.25 ± 49.81	224.36 ± 40.44	138.76 ± 52.76**	208.42 ± 42.34***	198.34 ± 77.62
Gastrocnemius						
ROS (U/mL)	582 ± 66.78	525.78 ± 98.76	504.56 ± 72.33	985.76 ± 95.78*	678.82 ± 62.74^****^	687.54 ± 178.72
3‐NT (ng/mL)	7.87 ± 3.62	8.18 ± 1.78	8.23 ± 1.98	14.72 ± 3.72**	8.32 ± 1.23***	10.50 ± 2.29***
MDA (nmol/mg)	5.86 ± 1.83	5.73 ± 1.50	5.18 ± 2.16	8.75 ± 2.76*	6.39 ± 1.15***	6.25 ± 1.67***
SOD (U/mg)	34.66 ± 7.89	36.45 ± 7.21	33.71 ± 10.44	20.45 ± 6.78*	30.13 ± 8.92***	27.54 ± 10.34
GSH‐Px (U/mg)	304.34 ± 45.34	342.25 ± 44.31	318.28 ± 60.62	154.43 ± 41.98**	289.32 ± 59.73***	280.25 ± 47.71***

*Note*: *n* = 8 per group.

Abbreviations: 3‐NT, 3‐nitrotyrosine; CK, creatine kinase; GSH‐Px, glutathione peroxidase; LDH, lactate dehydrogenase; MDA, malondialdehyde; ROS, reactive oxygen species; SOD, superoxide dismutase.

* *p* < 0.05 compared with control.

** *p* < 0.01 compared with control.

*** *p* < 0.05 compared with I/R.

^****^ *p* < 0.01 compared with I/R.

### 3.3 NBP alleviates I/R induced skeletal muscle injury in mice via activating Sirt1/Nrf2 signaling pathway

**FIGURE 2:**
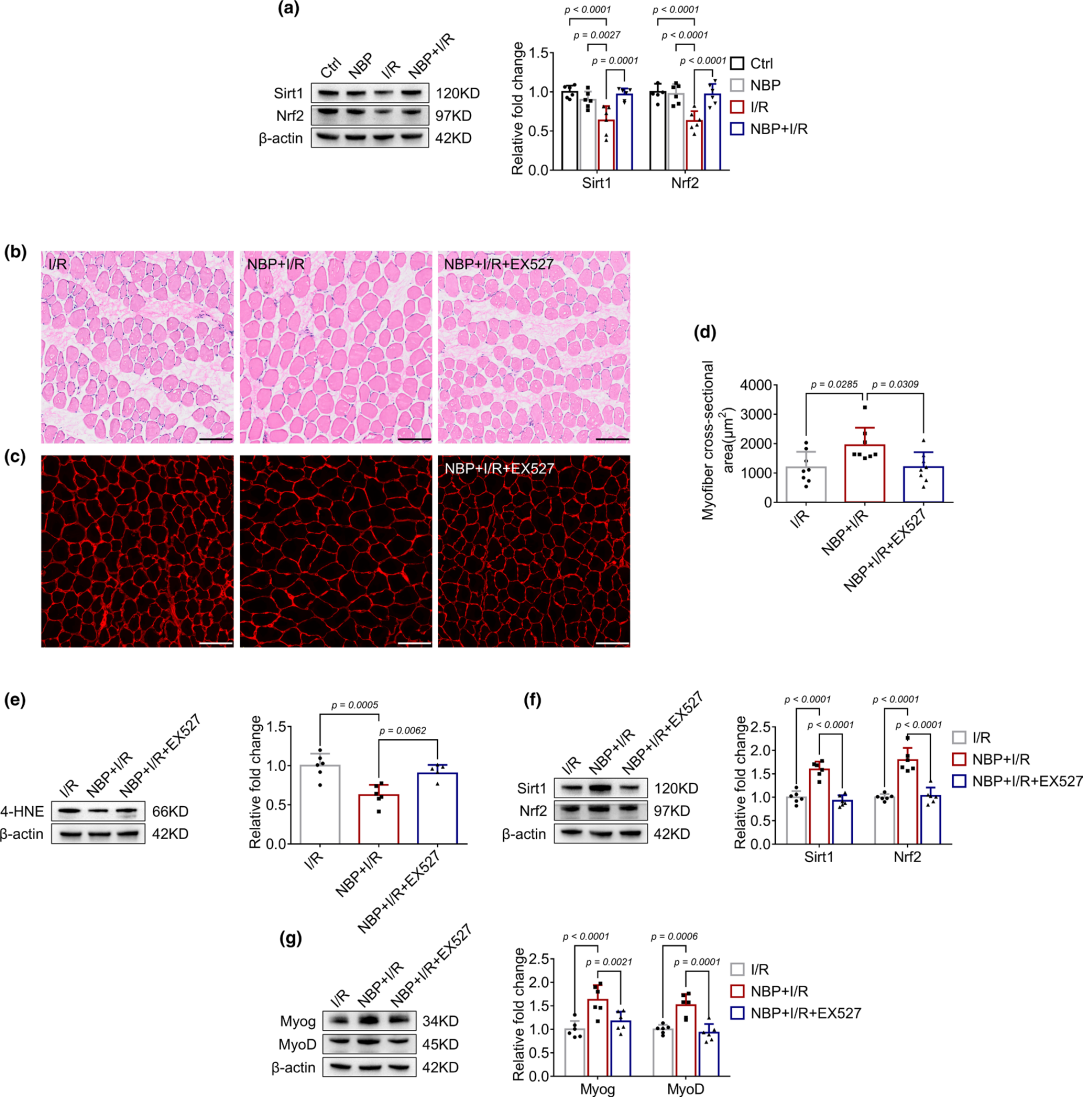
NBP activates Sirt1/Nrf2 signaling pathway in I/R‐induced skeletal muscle injury. (a) Western blot analysis in gastrocnemius muscle indicates the effects of NBP on Sirt1, Nrf2 expression. Data are presented as relative fold change to the control group (set to 1). *n* = 6 per group. (b–d) H&E staining and immunofluorescence staining of the gastrocnemius muscle indicate the extent of muscle fiber injury and changes in muscle fiber cross‐sectional area across different groups after EX527 treatment. *n* = 8 per group. Scale bar = 100 μm. A microscope with a 40× objective was used to capture the images. (e–g) Western blot analysis in gastrocnemius muscle indicates the effects of 4‐HNE, Sirt1, Nrf2, Myog and MyoD expressions after EX527 treatment. Data are presented as relative fold change to the I/R group. *n* = 6 per group.

Previous studies have demonstrated an important role of Sirt1/Nrf2 signal pathway in I/R‐induced oxidative stress (Wafi et al., 2019). In the present study, the expression of Sirt1 and Nrf2 were decreased in the gastrocnemius muscle after I/R procedure, whereas NBP treatment could preserve their expression (Figure 2a). To investigate whether Sirt1 was crucial for the protective effect of NBP, the Sirt1 inhibitor EX527 was applied. As shown in Figure 2b–d, the I/R process caused disruption of skeletal muscle fiber structure and a reduction in muscle fiber cross‐sectional area. NBP treatment alleviated I/R‐induced fiber irregularities and increased the gastrocnemius muscle fiber cross‐sectional area, whereas the administration of EX527 abolished the effects of NBP. Furthermore, we found that EX527 not only weakened the effects of NBP on oxidative stress biomarkers (Table 2), but also attenuated the downregulatory effect of NBP on 4‐HNE expression in I/R‐stimulated skeletal muscle (Figure 2e) and diminished the upregulatory effects of NBP on Sirt1 and Nrf2 expression (Figure 2f). In addition, EX527 also reversed the protective effects of NBP on Myog and MyoD expression (Figure 2g). These results indicated that NBP might protect skeletal muscle against I/R injury by activating Sirt1/Nrf2 signal pathway.

**TABLE 2:** The attenuation of oxidative stress by NBP in mice model of I/R‐induced skeletal muscle injury was inhibited by EX527.

Parameters	I/R	NBP + I/R	NBP + I/R + EX527
Gastrocnemius			
ROS (U/mL)	688.97 ± 72.11	498.77 ± 65.12*	748.78 ± 90.62
3‐NT (ng/mL)	33.78 ± 6.22	25.12 ± 5.61*	36.11 ± 8.42***
MDA (nmol/mg)	8.21 ± 2.79	3.31 ± 1.24*	7.56 ± 1.38***
SOD (U/mg)	26.92 ± 12.23	32.86 ± 6.24	34.24 ± 11.55
GSH‐Px (U/mg)	145.12 ± 41.36	218.62 ± 36.64*	162.56 ± 47.22***

*Note*: *n* = 8 per group.

Abbreviations: 3‐NT, 3‐nitrotyrosine; GSH‐Px, glutathione peroxidase; MDA, malondialdehyde; ROS, reactive oxygen species; SOD, superoxide dismutase.

* *p* < 0.05 compared with I/R.

*** *p* < 0.05 compared with NBP + I/R.

### 3.4 NBP ameliorates H/R‐induced C2C12 myotube injury by activating Sirt1/Nrf2 pathway

**FIGURE 3:**
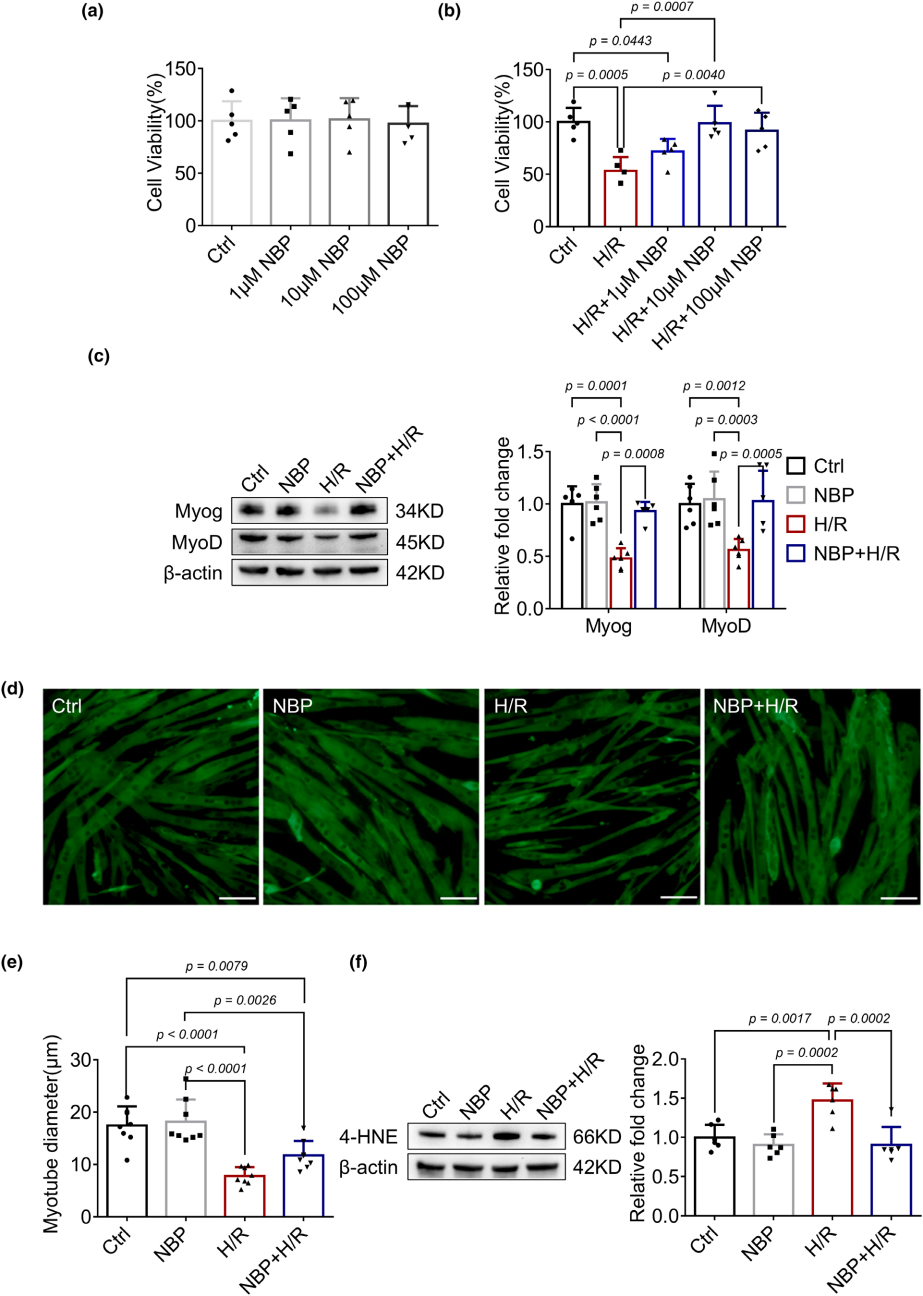
NBP alleviates H/R induced C2C12 myotube injury. (a) C2C12 myotubes were treated with different concentrations of NBP for 24 h and changes in myotubes viability were assessed. *n* = 5 per group. (b) C2C12 myotubes were pretreated with NBP at different concentrations for 24 h followed by H/R and changes in myotubes viability were assessed. *n* = 5 per group. (c) Myog and MyoD expressions were detected by Western blot in each group of C2C12 myotubes. Data are presented as relative fold change to the control group. *n* = 6 per group. (d, e) Representative immunocytochemistry staining of myotubes showed the changes in myotubes diameter in different groups. *n* = 8 per group. Scale bar = 50 μm. A microscope with a 40× objective was used to capture the images. (f) Western blot analysis in each group of C2C12 myotubes indicates the effects of NBP on 4‐HNE expression. Data are presented as relative fold change to the control group (set to 1). *n* = 6 per group.

**FIGURE 4:**
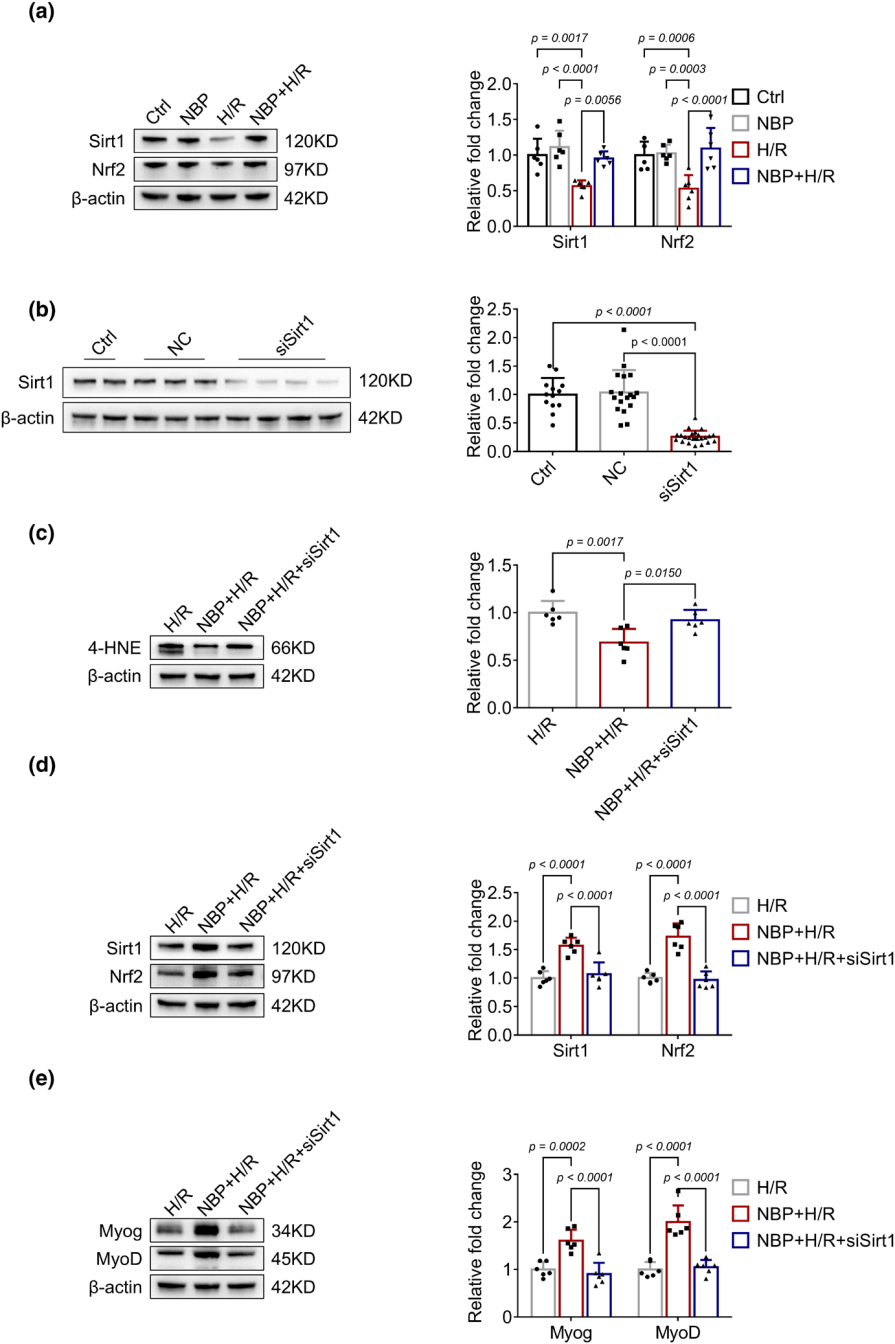
NBP activates Sirt1/Nrf2 pathway in H/R‐induced injury in C2C12 myotubes. (a) Sirt1 and Nrf2 expressions were detected by Western blot in each group of C2C12 myotubes. Data are presented as relative fold change to the control group. *n* = 6 per group. (b) The efficiency of siRNA targeting Sirt1. Data are presented as relative fold change to the control group. (c–e) The effects of NBP on the expression of 4‐HNE, Sirt1, Nrf2, Myog and MyoD after silencing of Sirt1. Data are presented as relative fold change to the H/R group. *n* = 6 per group.

To confirm the in vivo results, we further investigated the effects of NBP in H/R‐induced C2C12 myotubes injury model. Adding concentrations of 1, 10, and 100 μM of NBP to mature C2C12 myotubes does not decrease cell viability (Figure 3A). At concentrations of 10 and 100 μM, NBP can reduce cell death induced by H/R (Figure 3B). Consequently, this study has adopted 10 μM as the intervention dosage for NBP. As shown in Figure 3C, H/R stimulation caused decreased expression of Myog and MyoD as well as significant myotube atrophy (Figure 3D,E). C2C12 myotubes were pretreated with 10 μM NBP before C2C12 myotube H/R. As expected, NBP upregulated the expression levels of Myog and MyoD and alleviated myotube atrophy induced by H/R stimulation. Consistent with the animal results, H/R stimulation increased 4‐HNE levels (Figure 3F) and decreased Sirt1 and Nrf2 expression (Figure 4A), both of which were reversed by NBP treatment. Moreover, silencing Sirt1 with siRNA also dampened the downregulation of 4‐HNE (Figure 4C) and the upregulation of Nrf2, Myog, and MyoD (Figure 4D,E) induced by NBP. These results demonstrated that NBP might ameliorate H/R‐induced myotube injury through Sirt1/Nrf2 pathway.

## 4 DISCUSSION

In the present study, we showed that NBP treatment could alleviate I/R‐induced skeletal muscle injury both in vivo and in vitro. Mechanistically, the protective effects of NBP might be mediated, at least in part, through activating of the Sirt1/Nrf2 signaling pathway. Therefore, NBP might be a potential therapeutic agent for the treatment of I/R injury‐induced skeletal muscle damage.

Skeletal muscle is a high energetic organ which is sensitive to reperfusion injury after ischemia (Cheng et al., 2016). Reperfusion of ischemic tissue with oxygenated whole blood, increases early ischemic injury by release of ROS such as superoxide anions, hydroxyl radicals, and hydrogen peroxides (Zhang, Pan, et al., 2022). Excessive ROS breaks the redox balance which in turn results in I/R injury (Chen et al., 2022). Therefore, modulation of I/R‐associated oxidative stress by inhibiting ROS production seems to be a promising strategy to attenuate I/R injury (Wu et al., 2020). Our results showed that NBP treatment could reduce I/R‐induced oxidative stress and skeletal muscle injury. Our data are consistent with a recent study showing that NBP exerts antioxidant properties and mitigates tissue damage in skeletal muscle (Sun et al., 2022). However, the exact efficacy and safety of NBP in the treatment of patients with I/R‐induced skeletal muscle injury still need to be further investigated.

NBP has been well known as a synthetic chiral compound which has protective effects on ischemic stroke and neurodegenerative diseases (Chen et al., 2019). The underlying mechanisms mainly include alleviating oxidative stress, protecting mitochondrial function, reducing neuronal apoptosis, attenuating inflammatory response, and enhancing neurogenesis (Chen et al., 2019). Gong et al., 2019 found that NBP could attenuate acute lung injury by reducing oxidative stress via activating the Sirt1/Nrf2 signaling pathway. Consistently, our results also showed that I/R stimulation decreased the expression of Sirt1 and Nrf2, which was rescued by NBP treatment. Importantly, block of Sirt1 either by specific inhibitor or siRNA almost diminished the protective effects of NBP on I/R injury, suggesting that NBP may protect against I/R‐induced skeletal muscle injury via Sirt1/Nrf2 pathway. Further studies by using skeletal muscle‐specific Sirt1 or Nrf2 knockout mice will needed to elucidate the exact role of Sirt1/Nrf2 pathway in the therapeutic effects of NBP on I/R‐induced skeletal muscle injury. Besides, proteomic analysis may help to uncover other potential molecular mechanisms for the protective effects of NBP.

## 5 CONCLUSIONS

**FIGURE 5:**
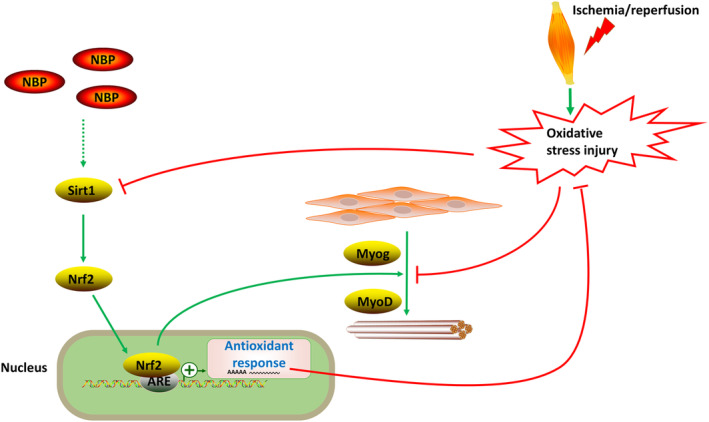
Schematic of the mechanism by which NBP attenuates I/R‐induced skeletal muscle injury. NBP may alleviate I/R‐induced skeletal muscle injury by activating the Sirt1/Nrf2 signaling pathway and promoting the expression of Myog and MyoD, thereby exerting a protective effect during the progression of I/R‐induced skeletal muscle damage.

In summary, our findings demonstrate that NBP may alleviate I/R‐induced skeletal muscle injury via activating Sirt1/Nrf2 pathway (Figure 5). However, long‐term prospective cohort studies are still needed to confirm the protective effects of NBP on patients with I/R‐induced skeletal muscle injury.

## FUNDING INFORMATION

This work was supported by grants from the National Key Research and Development Plan of China (No. 2020YFC2008505 to Xiang Lu), the National Natural Science Foundation of China (No. 81970217 to Wei Gao), the Jiangsu Commission of Health (Nos. LR2022004 and LKZ2023005 to Wei Gao), and Commissioned Project of the 2023 Special Fund for Elderly Appropriate Technology by the Jiangsu Geriatrics Society (No. JGS2023WTSY05 to Peng Lu).

## CONFLICT OF INTEREST STATEMENT

None of the authors has any conflict of interest to disclose.

## ETHICS STATEMENT

All of the animal experiments were carried out under the approval of the Animal Ethics Committee of Nanjing University (permit number: IACUC‐1905027) and complied with the National Institutes of Health guide for the care and use of laboratory animals (NIH Publications No. 8023, revised 1978).

## Data Availability

All data and materials used for this study are displayed or can be displayed upon request.
